# Patient reported outcome measure thresholds: The next step in the management of polycystic liver disease

**DOI:** 10.1002/ueg2.12394

**Published:** 2023-05-01

**Authors:** Renée Duijzer, Tom J. G. Gevers

**Affiliations:** ^1^ Department of Gastroenterology and Hepatology Radboudumc Nijmegen The Netherlands; ^2^ Department of Gastroenterology and Hepatology Maastricht University Medical Centre Maastricht The Netherlands; ^3^ Nutrim School for Nutrition and Translational Research in Metabolism Maastricht University Maastricht The Netherlands

The paper entitled “The polycystic liver disease‐specific questionnaire POLCA identifies patients in need for therapy: a multi‐centric prospective study” by Billiet et al. highlights an important issue.^1^ Patients with severe polycystic liver disease (PLD) suffer from pain, mobility restrictions, sarcopenia as a result of stomach compression and psychological complaints. The management of these patients is directed to mitigate these symptoms, most often through cyst volume reducing therapies.

PLD is a rare genetic inherited disorder in which increased proliferation of cystic cholangiocytes contributes to hepatic cystogenesis.^2^ Although severe hepatomegaly does not affect liver function, mechanical symptoms can be an indication for therapy and even liver transplantation (LT) in advanced cases.^3^ Therefore, the pillars of management in PLD focus on symptom relief and improvement of Health‐related quality of life.

This paper highlights an important effort to determine relevant clinical thresholds to aid decision‐making in the management of PLD using patient reported outcome measures (PROMs). The PROM in question is the PLD‐complaint‐specific assessment (POLCA), which consists of several components, including a Severity of Perceived Illness (SPI) score. In this prospective observational study, the SPI score was found to be an adequate indicator of the need to start somatostatin analogs (SA) or LT in patients with severe PLD. In addition, a significant correlation was found between the POLCA SPI score, height adjusted total liver volume (hTLV) and changes in hTLV over time.

PROMs measure the subjective elements of a patient's condition, evaluate the burden of disease or treatment from patients' perspectives, and are pivotal in the treatment of PLD as they allow clinicians to measure relevant management endpoints.^4^ Both the Food and Drug Administration and the European Medicines Agency have acknowledged PROMs as a measure of treatment efficacy and also encourage implementation in clinical trials.^5^ By actively involving patients in the assessment and management of their condition, healthcare providers obtain a more comprehensive understanding of their patients complaints and also enable them to take an active role in their care.

Currently, two PLD‐complaint‐specific validated PROMs are available for PLD, the POLCA described in this article and the PLD‐Q.^6^, ^7^ A review by the authors of this paper showed that these symptom specific PROMs are increasingly applied. However, the lack of treatment thresholds contributes to the present heterogeneity in the application of PROMs.^8^ A more systematic and uniform establishment and implementation of PROM thresholds would assist healthcare providers in disease monitoring, treatment initiation and therapy evaluation in PLD. An important first step has been taken by determining POLCA thresholds that indicate the need for SA therapy (SPI > 14) or LT (SPI > 18).

However, it is important to recognize that relying solely on PROM thresholds may have pitfalls. Thresholds are designed to categorize patients into discrete levels of severity. This approach may be faulted if thresholds are affected by factors not related to PLD. For example, PLD patients who also suffer from functional gastrointestinal tract disorders might have increased PLD‐complaint assessments, but probably do not respond well to volume‐reducing therapies. Therefore, it remains important to keep a certain threshold for hTLV in mind when starting therapy. Additionally, other patient factors, such as the need for renal transplantation because of polycystic kidney disease, could guide a decision towards LT instead of surgical therapy. Finally, these disease specific PROMS are not validated to measure sarcopenia, which plays an important role in the decision to transplant, as the authors have already mentioned in their discussion.

PROM thresholds can also be applied to measure the efficacy of PLD treatments. Billiet et al. found a significant correlation between the POLCA SPI score and changes in hTLV, but this does not automatically indicate a significant clinical effect for individual patients. If a minimal important change could be established, a minimal within‐person change over time in which patients perceive themselves as a relevant change in symptoms, it would provide better insight into assess treatment effect.^9^

In conclusion, the established POLCA thresholds that give an indication when to start PLD treatment, and similar thresholds are expected to be available for the PLD‐Q shortly. As a next step, we should strive for a more systematic implementation of these PROM thresholds in clinical practice to guide the initiation of PLD therapies. However, we must be careful to not focus solely on PROM thresholds in the decision process but also take the clinical phenotype and patient specific factors into account.

## FUNDING INFORMATION

The authors received no financial support for the research, authorship, and/or publication of this article.

## CONFLICT OF INTEREST STATEMENT

The authors have no conflicts of interest to declare.

## Data Availability

Data sharing is not applicable to this article as no new data were created or analyzed in this study.
